# Der „Professional Ear User“ – Implikationen für die Prävention, Diagnostik und Therapie von Ohrerkrankungen

**DOI:** 10.1007/s00106-022-01235-0

**Published:** 2022-10-21

**Authors:** David Bächinger, Raphael Jecker, Jean-Christoph Hannig, Andreas Werner, Horst Hildebrandt, Michael Eidenbenz, Martin Kompis, Tobias Kleinjung, Dorothe Veraguth

**Affiliations:** 1 Klinik für Ohren‑, Nasen‑, Hals und Gesichtschirurgie, Universitätsspital Zürich, Frauenklinikstraße 24, 8091 Zürich, Schweiz; 2 Universität Zürich, Zürich, Schweiz; 3 Tonmeister/Departement Musik, Zürcher Hochschule der Künste, Zürich, Schweiz; 4 Klavierbau und Konzerttechnik, Werkstatt für Klaviere und Flügel, Musik Hug AG, Bülach, Schweiz; 5 Departement Musik, Zürcher Hochschule der Künste, Zürich, Schweiz; 6 Musikphysiologie, Musik- und Präventivmedizin, Departement Musik, Zürcher Hochschule der Künste, Zürich, Schweiz; 7 Direktion, Departement Musik, Zürcher Hochschule der Künste, Zürich, Schweiz; 8 Universitätsklinik für Hals, Nasen- und Ohrenkrankheiten, Kopf- und Halschirurgie, Inselspital, Universitätsspital Bern, Bern, Schweiz

**Keywords:** Ohrgesundheit, Lärm, Gehörschutz, Vorsorge, Musik, Hearing health, Noise, Hearing protection, Prevention, Music

## Abstract

**Hintergrund:**

Ein vollständig intaktes Hörvermögen ist zentral für die Ausübung verschiedener Berufe wie Instrumentenbaumeister, Musiker, Tonmeister sowie für weitere Berufsgruppen ohne Bezug zu Musik wie beispielsweise Sonar-Techniker. Für Personen all dieser Berufsgruppen schlagen wir in Analogie zum „Professional Voice User“ den Begriff „Professional Ear User“ (PEU) vor. PEU haben spezielle Anforderungen an ihre Ohrgesundheit, da sie über eine überdurchschnittliche auditive Wahrnehmungsfähigkeit verfügen, von der sie beruflich abhängig sind.

**Fragestellung:**

Die vorliegende narrative Übersichtsarbeit hat zum Ziel, die sich daraus ergebenden speziellen Aspekte der Prävention, Diagnostik und Therapie von Ohrerkrankungen bei PEU zusammenzufassen.

**Ergebnisse und Schlussfolgerung:**

Die Prävention von Hörstörungen und weiteren Ohrerkrankungen umfasst den Schutz vor zu hohen Schallpegeln, die Vermeidung von Ototoxinen oder Nikotin sowie die korrekte Durchführung einer Gehörgangsreinigung. Die Abklärung von Hörstörungen kann sich bei PEU herausfordernd gestalten, da subklinische, jedoch einschränkende Veränderungen des Hörvermögens mit konventionellen audiometrischen Methoden nicht zuverlässig objektiviert werden können. Schließlich kann das Vorliegen einer Ohrerkrankung bei einem PEU Therapieentscheidungen beeinflussen. Weiter muss bei PEU auch eine hohe Wachsamkeit bezüglich nichtorganischer Ohrerkrankungen bestehen. Abschließend werden Möglichkeiten diskutiert, um bei PEU eine umfassende Ohrgesundheit im Rahmen eines edukativen Programms zu fördern und mittels einer spezialisierten ohrenärztlichen Sprechstunde zu erhalten. Im Gegensatz zu bestehenden Konzepten ist der Fokus dabei auf die Gesamtheit der Berufsgruppen gerichtet, welche in professionellem Rahmen speziell von der Ohrgesundheit abhängig sind. Außerdem soll der Schwerpunkt hierbei nicht nur auf Hörstörungen und deren Prävention, sondern auch auf der Erhaltung einer ganzheitlichen Ohrgesundheit liegen.

## Abstract (en)

**Background:**

Perfect hearing is crucial to the practice of various professions, such as instrument makers, musicians, sound engineers, and other professions not related to music, such as sonar technicians. For people of these occupational groups, we propose the term “professional ear user” (PEU) in analogy to “professional voice user”. PEUs have special requirements for their hearing health, as they have well-known above-average auditory perceptual abilities on which they are professionally dependent.

**Objective:**

The purpose of this narrative review is to summarize selected aspects of the prevention, diagnosis, and treatment of ear disorders in PEUs.

**Results and conclusion:**

Prevention of hearing disorders and other ear diseases includes protection from excessive sound levels, avoidance of ototoxins and nicotine, and a safe manner of cleaning the outer auditory canal. Diagnosing hearing disorders in PEUs can be challenging, since subclinical but relevant changes in hearing cannot be reliably objectified by conventional audiometric methods. Moreover, the fact that a PEU is affected by an ear disease may influence treatment decisions. Further, physicians must be vigilant for non-organic ear diseases in PEUs. Lastly, measures to promote comprehensive ear health in PEUs as part of an educational program and to maintain ear health by means of a specialized otolaryngology service are discussed. In contrast to existing concepts, we lay the attention on the entirety of occupational groups that are specifically dependent on their ear health in a professional setting. In this context, we suggest avoiding a sole focus on hearing disorders and their prevention, but rather encourage the maintenance of a comprehensive ear health.

## Hintergrund

Eine Vielzahl von Personen sind aus professionellen Gründen auf ein vollständig intaktes Hörvermögen angewiesen. Dazu zählen z. B. Musiker/‑innen, Tonmeister/‑innen, oder Klaviertechniker/‑innen. Während für andere Berufsgruppen wie beispielsweise den „Professional *Voice* User“ (Sänger/‑innen, Sprecher/‑innen, Schauspieler/‑innen u. a.) spezielle Empfehlungen und Richtlinien für die Prävention, Diagnostik und Therapie von Stimmstörungen existieren [76, 115], ist die Gruppe der professionell auf ein hochleistungsfähiges Hörorgan angewiesenen Personen kaum definiert. Entsprechend gibt es für diese Gruppe wenig Literatur und Empfehlungen zur Prävention, Diagnostik und Therapie von Hörstörungen bzw. Ohrerkrankungen im Allgemeinen. In Analogie zum „Professional Voice User“ schlagen wir die Bezeichnung „Professional Ear User“ (PEU) vor, welche nicht nur Musiker/‑innen, sondern alle in einem professionellen Rahmen stark auf ein uneingeschränktes Hörvermögen angewiesene Personen einschließt. Dazu gehören u. a. Instrumentalmusiker/‑innen, Sänger/‑innen, Dirigenten/‑innen, Komponisten/‑innen, Musikwissenschaftler/‑innen, Instrumententechniker/‑innen und -baumeister/‑innen, Musikproduzenten/‑innen und Tonmeister/‑innen bzw. Personen in Ausbildung zu diesen Berufen. Weiter finden sich auch PEU ohne Bezug zu Musik, beispielsweise Logopäden/‑innen, Sonar-Techniker/‑innen^1^ [70] oder Ornithologen/‑innen [30, 57]. Am Beispiel von Musikern wurde gezeigt, dass PEU über eine außergewöhnliche auditive Wahrnehmungsfähigkeit verfügen, z. B. eine überdurchschnittliche Fähigkeit zur Tonhöhenunterscheidung oder zum frequenzselektiven Hören in komplexer akustischer Umgebung [58, 87]. Diese überdurchschnittliche auditive Wahrnehmungsfähigkeit spiegelt sich auch in neuroplastischen Veränderungen wider, die mittels moderner bildgebender Methoden sichtbar gemacht werden können [40, 114]. Vor diesem Hintergrund erscheint es plausibel, dass die berufliche Tätigkeit von PEU bereits durch eine diskrete Hörstörung drastisch gestört sein kann [46], wobei eine solche Hörstörung in der klinischen Audiometrie gar nicht abgebildet sein oder noch innerhalb der Normalwerte liegen kann. Da zusätzlich insbesondere Erkrankungen des Innenohrs kaum kausal behandelt werden können, kommt der Prävention von Ohrerkrankungen bei PEU im Sinne einer Verhinderung bzw. Verminderung jeglicher schädigenden Einflüsse auf das Hörorgan eine eminente Rolle zu, sodass auch „subklinische“ Schäden am Hörorgan nach Möglichkeit vermieden werden [46]. Hierzu stellt sich weiter die Frage nach der Notwendigkeit sowie Regelmäßigkeit von ohrenärztlichen bzw. audiometrischen Vorsorgeuntersuchungen. In der audiometrischen Abklärung von PEU müssen insbesondere die Bedeutung und Grenzen des konventionellen Reintonaudiogramms in Betracht gezogen werden. Entsprechend muss die diagnostische und prognostische Wertigkeit weiterführender (audiometrischer) Untersuchungen in dieser speziellen Population besprochen werden. Falls es schließlich tatsächlich zu akuten Ohrerkrankungen wie Tubenventilationsstörungen, einer akuten Otitis media oder einem Hörsturz kommt, kann die Tatsache, dass ein PEU betroffen ist, Einfluss auf Therapieentscheidungen haben. Bei Auftreten von chronischen Ohrerkrankungen, z. B. einer Schwerhörigkeit oder Tinnitus, ist bei PEU das Wissen um deren berufliche Tätigkeit ebenfalls zentral. So wirkt eine Schwerhörigkeit bei PEU stark stigmatisierend und stellt bei einer eventuellen Hörgeräteversorgung spezielle Herausforderungen an den Hörgeräteakustiker [17, 113].

Die vorliegende narrative Überblicksarbeit soll aufzeigen, wie spezielle Voraussetzungen und Anforderungen von PEU Prävention, Diagnostik und Therapie von Ohrerkrankungen beeinflussen. Daraus ergeben sich verschiedene Vorschläge und Empfehlungen mit dem Fokus Ohrerkrankungen, insbesondere Hörstörungen, bei PEU zu verhindern bzw. adäquat zu therapieren. Diese Aspekte sollen auch als Vorschlag gelten, was PEU im Rahmen der Berufsausbildung im Sinne eines edukativen Programms über die Ohrgesundheit vermittelt werden soll und wie die ohrenärztliche Betreuung von PEU ablaufen kann. Dies geschieht im Bestreben, eine ganzheitliche Ohrgesundheit bei PEU so zu schützen, erhalten und wiederherzustellen, dass eine Berufsausübung in Abhängigkeit von einem möglichst optimal funktionierenden Hörorgan gewährleistet werden kann.

## Prävention von Ohrerkrankungen

Grundlegende Kenntnisse der Anatomie des Ohrs, der Physiologie des Hörorgans sowie von häufigen Ohrerkrankungen sind zentral für ein Verständnis jeglicher präventiver, diagnostischer und therapeutischer Maßnahmen. Entsprechend sollen PEU möglichst früh im Laufe der Berufsfindung der Aufbau und die Funktionsweise des Ohres und des Hörorgans, sowie deren wichtigste Erkrankungen nahegelegt werden.

Die Prävention von Ohrerkrankungen bei PEU zielt insbesondere darauf ab, vorübergehende und bleibende Hörschäden zu verhindern. Dabei muss bei PEU ein Wissen über vermeidbare gehörschädigende Einflüsse bestehen, welche insbesondere Lärm und ototoxische Substanzen umfassen. Weiter können unter Umständen kardiovaskuläre Risikofaktoren, insbesondere der Nikotinabusus, schädlichen Einfluss auf das Gehör haben. Schließlich können bleibende Hörstörungen durch Erkrankungen des äußeren Ohres, des Mittel- sowie des Innenohres entstehen, deren rechtzeitige und adäquate Therapie deshalb insbesondere auch bei PEU eine besondere Rolle zukommt (s. Abschnitt „Spezielle Aspekte von Ohrerkrankungen und Therapie“).

### Lärm

Lärm ist der mit Abstand bedeutendste beeinflussbare Risikofaktor für eine Schwerhörigkeit. Seit Jahrzehnten ist gut bekannt, dass eine übermäßige Lärmexposition nach wenigen Stunden zu einer Schädigung der schalldetektierenden Haarzellen im Innenohr führt, was wiederum eine permanente Anhebung der Hörschwelle bewirkt [66]. Bis vor gut zehn Jahren existierte das Dogma, dass Lärm primär Haarzellen schädigt. In der Umkehr wurde geschlossen, dass eine Lärmschädigung, welche keine permanente Hörschwellenerhöhung verursacht, ohne einen Haarzellverlust einhergeht und daher gutartig ist [72]. Diese Annahme liegt auch den aktuellen Kriterien für das Schädigungsrisiko von Lärm am Arbeitsplatz zugrunde [3, 72]. In den letzten Jahren hat jedoch Forschung in Tiermodellen gezeigt, dass auch eine Lärmbelastung mit einer nur vorübergehenden Hörschwellenerhöhung zwar nicht zu einem Haarzellschaden, jedoch zu einer Schädigung des Hörnervs führen kann [61]. Auch bei Menschen wurde gezeigt, dass beispielsweise bereits eine Belastung von 88 dB (A) über 20 min zu einer temporären Hörschwellenverschiebung von bis zu 40 dB führt [23]. Dabei kommt es zu einem Verlust der Kontakte zwischen Hörnervenfasern und Haarzellen (sog. cochleäre Synaptopathie) [67]. Eine cochleäre Synaptopathie kann vermutlich zu Tinnitus, Hyperakusis und einer verminderten Sprachverständlichkeit führen, auch wenn sich im konventionellen Reintonaudiogramm eine Normakusis findet (sog. „hidden hearing loss“) [59, 67]. Somit scheint ein konsequenter und großzügig eingesetzter Gehörschutz insbesondere bei PEU angezeigt, und zwar möglicherweise auch bei tieferen Schallpegeln als gesetzlich vorgeschrieben [67].

Bereits unverstärkte Musik, wie sie beispielsweise beim individuellen Üben oder Stimmen von Instrumenten erklingt, kann schädigende Schallpegel aufweisen (eine entsprechende Schallpegeltabelle findet sich unter [122]). In diesem Zusammenhang gibt es in der Musikermedizin seit Jahrzehnten eine zunehmende Anzahl von Studien, welche eine hohe Prävalenz von Hörschäden bei professionellen Musikern zeigen, was eine vermehrte Anwendung von gehörschützenden Maßnahmen bei Musikern sowohl in Orchestern als auch bei Konzerten mit verstärkter Musik zur Folge hatte [31, 83, 95]. Musiker sind aber nur eine von vielen gefährdeten Berufsgruppen, welche schädigenden Schalldruckpegeln ausgesetzt und gleichzeitig in hohem Maß auf ein funktionierendes Hörorgan angewiesen ist. Deshalb sollten die Grundsätze zum Gehörschutz für Musiker auch auf andere PEU angewendet werden, z. B. Tonmeister oder Klavierbaumeister und andere Instrumentenbauer.

Grundsätzlich sollten neben Gehörschutzmitteln jegliche Maßnahmen zur Verringerung der Schallpegel wahrgenommen werden. Hierzu gehören eine Vergrößerung des Abstands zur Schallquelle, eine Optimierung der Raumakustik sowie der Einsatz von Reflektoren und Schallschirmen. Weiter sollten im beruflichen Alltag wählbare Schallpegel wie z. B. Abhörpegel bewusst zurückhaltend auf ein nicht schädigendes Maß limitiert werden [70].

Am Beispiel von Musikern ist bei PEU gut bekannt, dass insgesamt eine starke Skepsis gegenüber einem Gehörschutz besteht und Gehörschutzmittel beispielsweise bei Orchestermusikern eher selten angewendet werden [31, 64, 118]. Auch wenn sich etwa zwei Drittel von Orchestermusikern um ihr Gehör sorgen [91], finden über 80 % die Anwendung eines Gehörschutzes schwierig oder unmöglich [80]. Neben der Einschränkung des Klangerlebens hat dies sicherlich auch mit einer stigmatisierenden Wirkung eines äußerlich sichtbaren Gehörschutzes zu tun. Weiter scheinen PEU die tatsächlich erlebten Schallpegel über die Zeit zu unterschätzen, weshalb eine Messung der Schallpegel am Tätigkeitsort von PEU dringend empfohlen bzw. in gewissen Situationen sogar rechtlich vorgeschrieben ist (vgl. hierzu beispielsweise die europäische Arbeitsschutzrichtlinie „Lärm“ 2003/10/EG oder die Schweizer Verordnung über die Verhütung von Unfällen und Berufskrankheiten, Artikel 34 Lärm und Vibrationen). In jedem Fall besteht bei einem vorübergehenden Tinnitus, Ohrdruckgefühl und/oder Dysakusis nach einer Schallexposition der Verdacht auf eine schädliche Schallexposition, was weitere Schritte zur Bestimmung der Schallexposition und einer audiometrischen Abklärung des PEU zur Folge haben sollte [46].

Informationen über die Bedeutung und Anwendung eines konsequenten Gehörschutzes bei schädigenden Schallpegeln sind zentral bei PEU. In diesem Kontext ist speziell die Aufklärung über konkrete Typen von Gehörschutzmitteln wichtig [91]. Die Anforderungen an einen Gehörschutz bei PEU sind sowohl hinsichtlich des Tragekomforts als auch des akustischen Dämpfungsprofils hoch. Durch den Verschluss des Gehörgangs durch den Gehörschutz kommt es zur Dämmung des Schalls, welcher auf das Trommelfell trifft. Begleitend werden durch die Okklusion eigene Körpergeräusche verstärkt wahrgenommen (sog. Okklusionseffekt) [33, 44]. Zusätzlich wird der Resonanzraum des Gehörgangs aufgehoben, womit eine selektive Verstärkung der Frequenzbänder um etwa 3 kHz wegfällt [25]. Schließlich dämmen selbst individuell angepasste und nur wenig dämmende (< 10 dB) Gehörschutzmittel über das Frequenzspektrum unterschiedlich stark [2]. Somit kommt es insgesamt zu einer Veränderung des Höreindrucks sowohl durch einen verminderten Schallpegel als auch durch eine Veränderung des Frequenzspektrums. Dies kann die Beurteilung einer Schallinformation beeinflussen, was für PEU besonders schwerwiegend sein kann. Weiter stellt sich die Frage, ob die Benutzung von Gehörschutzmitteln die spektralen Eigenschaften produzierter Musik verändert. Hierzu existiert eine kontroverse Studienlage, wobei eine aktuelle Studie mit individuell angepassten Gehörschutzmitteln mit gleichmäßigen Dämmungseigenschaften keinen Einfluss auf die Klangfarbe oder dynamische Kontrolle bei Instrumentalmusikern fand [60, 106]. Bei Neuanschaffung eines Gehörschutzes ist mit einer Angewöhnungszeit von mehreren Wochen zu rechnen [128]. Auch das Einsetzen des Gehörschutzes einige Stunden vor der eigentlichen Schallexposition kann den Höreindruck natürlicher erscheinen lassen.

Als gängiges Gehörschutzmittel sind für PEU aufgrund des Schalldämmungsprofils und des Tragekomforts primär individuell angepasste Gehörschutz-Otoplastiken empfohlen [128]. Eine im Rahmen der Berufsausbildung durchgeführte, frühzeitige Information über mögliche Gehörschutzmittel, eine Hilfestellung bei der Auswahl sowie finanzielle Unterstützung zum Erwerb der kostspieligen Gehörschutzmittel könnte die Akzeptanz und Anwendungshäufigkeit erhöhen [91]. Eine neuere Entwicklung sind aktive Gehörschutzsysteme [43, 81]. Bei unverstärkter Musik kann beispielsweise ein passiver Gehörschutz mit einer aktiven Komponente nach dem Prinzip eines Hörgeräts mit Mikrofon, Prozessor und Lautsprecher kombiniert werden [81]. Somit kann die spektrale Information des Schalls besser erhalten und die Dämpfung bzw. Verstärkung des Schalls an den Schallpegel adaptiert werden [81]. Im Fall von verstärkter Musik kann ein solches System zusätzlich mit einem Im-Ohr-Monitor verbunden werden [43]. Für eine umfassende Beratung zu Gehörschutzmitteln sollen PEU an einen erfahrenen Hörgeräteakustiker verwiesen werden. Bei individuell angefertigten Gehörschutz-Otoplastiken muss dabei beachtet werden, dass nach der individuellen Anfertigung die Schutzwirkung nachgewiesen und periodisch überprüft werden muss (vgl. hierzu auch [130]).

Neben der beruflichen Schallexposition von PEU soll auch die Lärmbelastung im Rahmen der Freizeit beachtet werden [70]. So fand sich in einer kleinen Studie an Tonmeistern bei einer Mehrheit der Befragten eine erhebliche Lärmbelastung in der Freizeit [79]. Solche Lärmbelastungen sollen nach Möglichkeit vermieden werden, da dies unter anderem mit dem empfohlenen (wenn auch oft schwierig einzuhaltenden) Ruheintervall von zwölf bis vierundzwanzig Stunden zwischen den oft mehrstündigen beruflichen Schallexpositionen interferiert [46, 83].

Während ein konsequent und großzügig eingesetzter Gehörschütz bei PEU zentral ist, können übermäßig eingesetzte Gehörschutzmittel auch negative Auswirkungen mit sich bringen. Insbesondere bei ängstlich-vermeidenden Personen kann ein zu ausgeprägt angewandter Gehörschutz zu einem Teufelskreis aus Überprotektion, Hyperakusis und Phonophobie führen [8, 52, 74]. Zusätzlich erhöht das Tragen von Gehörschutzmitteln im Gehörgang die Zerumenproduktion, was eine regelmäßige Gehörgangsreinigung nötig machen kann (s. Abschnitt „Cerumen obturans“). Nichtsdestoweniger ist ein konsequenter Gehörschutz bei PEU die wichtigste Schutzmaßnahme für das Gehör.

### Ototoxische Substanzen

Ototoxische Substanzen sind neben Lärm eine der häufigsten Ursachen für eine sensorineurale Schwerhörigkeit [50]. Weit über 100 gängige Medikamente besitzen ototoxische Eigenschaften, u. a. Aminoglykoside, Zytostatika, Salicylate, Schleifendiuretika oder Antimalariamittel [54, 78, 97, 98]. Die Folgen der ototoxischen Wirkung können Tinnitus und/oder eine Schwerhörigkeit sein. Die klassischen, stark ototoxischen Substanzen wie z. B. Aminoglykoside oder Zytostatika werden jedoch zurückhaltend und in der Regel nur bei vitaler Indikation eingesetzt. Daneben gibt es aber auch eine Reihe von (teilweise rezeptfreien) ototoxischen Medikamenten, welche gelegentlich unkritisch eingesetzt werden und deren Ototoxizität möglicherweise unterschätzt oder wenig bekannt ist. Insbesondere bei PEU kann aber auch eine diskrete oder vorübergehende ototoxische Wirkung gravierende Folgen haben.

Ein wichtiges ototoxisches Medikament ist Acetylsalicylsäure, welches zur Therapie von Entzündungen und Schmerzen eingesetzt wird. Bei Einnahme von Acetylsalicylsäure können – in der Regel vorübergehend – ein Tinnitus, eine Schwerhörigkeit sowie eine Dysakusis auftreten, wobei das Risiko mit Zunahme der Dosis steigt [12]. Acetylsalicylsäure wird aufgrund des Nutzen-Risiko-Profils als zunehmend obsoletes Analgetikum betrachtet [68], weshalb von einer selbstständigen Einnahme von Acetylsalicylsäure bei PEU zur akuten Therapie von Schmerzen abgeraten werden muss. Neben Acetylsalicylsäure haben auch andere nichtsteroidale Antirheumatika gelegentlich ototoxische Nebenwirkungen, also z. B. Ibuprofen, Diclofenac, Naproxen oder Mefenamin [62]. Diese Nebenwirkungen scheinen aber weniger ausgeprägt zu sein als bei Acetylsalicylsäure [62]. Das Analgetikum Paracetamol weist vermutlich keine ototoxische Wirkung auf, weshalb dieses Medikament also auch hinsichtlich der ototoxischen Nebenwirkungen bei PEU primär zur Schmerzlinderung empfohlen werden kann.

Die Liste möglicher ototoxischer Medikamente ließe sich beliebig verlängern und kann hier nicht abschließend besprochen werden. Bei PEU soll jedenfalls das Bewusstsein für eine hohe Wachsamkeit bezüglich bekannter ototoxischer Nebenwirkungen bei Verschreibung von Medikamenten geschärft werden. Dabei ist allerdings auch zu beachten, dass gerade Tinnitus häufig als mögliche, wenig spezifische Nebenwirkung in Beipackzetteln aufgeführt ist und nicht immer einer ototoxischen Nebenwirkung entsprechen muss. Weiter soll die Aufklärung von PEU über gängige ototoxische Medikamente auch Hinweise auf alternative, zu bevorzugende Substanzen beinhalten.

### Nikotin

Epidemiologische Untersuchungen haben gezeigt, dass Rauchen mit einem erhöhten Risiko für Hörverlust und Tinnitus verbunden ist [13, 19, 24, 109]. Hierbei spielt wahrscheinlich sowohl eine Beeinträchtigung der Gefäßversorgung der Cochlea als auch eine direkte Cochleotoxizität eine Rolle [120]. Weiter wurde gezeigt, dass sich ein Rauchstopp positiv auf das Hörvermögen auswirkt, sodass sich dieses nach einem Rauchstopp nicht mehr vom Hörvermögen von Personen unterscheidet, welche nie geraucht haben [20, 38]. Entsprechend ist – auch aufgrund der unzähligen weiteren positiven Auswirkungen auf die Gesundheit – die Sistierung jeglichen Tabakkonsums bei PEU anzustreben.

### Diverse Risikofaktoren

Neben dem Nikotinabusus prädisponieren weitere kardiovaskuläre Risikofaktoren (Hypercholesterinämie, Diabetes mellitus, Adipositas) für eine Schwerhörigkeit. Dabei finden sich Assoziationen sowohl zu einem schnelleren Fortschreiten einer Altersschwerhörigkeit als auch zum Hörsturz [1, 39, 82, 90]. Umgekehrt findet sich ein positiver Zusammenhang zwischen der kardiovaskulären Gesundheit und dem Gehör, wobei Hinweise bestehen, dass sich die Prävention von kardiovaskulären Risikofaktoren auch positiv auf das Gehör auswirkt [20, 38]. Bei PEU mag zusätzlich neben der Prävention von kardiovaskulären Ereignissen die mögliche Prävention von Hörstörungen eine Zusatzmotivation zur Kontrolle und eventuellen Reduktion von kardiovaskulären Risikofaktoren sein. Schließlich kann auch Alkohol die Hörleistung insbesondere vorübergehend deutlich beeinträchtigen [51, 55, 107]. PEU sollten daher bei der Berufsausübung auf jeglichen Alkoholkonsum verzichten.

Schließlich gilt es bei allen Risikofaktoren zu bedenken, dass deren Kumulation zu einer überproportionalen Verstärkung der jeweiligen schädigenden Einflüsse führen kann, sodass z. B. die Einnahme von Acetylsalicylsäure und eine gleichzeitige Lärmexposition zu einem erhöhten Risiko für einen bleibenden Tinnitus führen [28].

## Screening und Diagnostik von Ohrerkrankungen

Zur Frage ob und – wenn ja – in welchen Abständen PEU einem Hörscreening bzw. einer allgemeinen ohrenärztlichen Untersuchung unterzogen werden sollten, gibt es nur wenige Empfehlungen. In jedem Fall sollten vorgeschriebene Untersuchungen in Anspruch genommen werden (s. Abschnitt „Lärm“). Regelmäßige audiometrische Untersuchungen im Abstand von ein bis zwei Jahren sind in jedem Fall wünschenswert, wobei die Intervalle an das individuelle Risiko angepasst werden sollen [31, 46]. Bereits zu Beginn der Ausbildung bzw. der Berufstätigkeit sollte das Gehör mindestens mittels eines Reintonaudiogramms dokumentiert werden [91], wobei die Knochenleitungsschwelle unabhängig von der Luftleitungsschwelle immer mitbestimmt werden soll. So wie zur Bewerbung auf verschiedene Ausbildungsgänge für „Professional *Voice* User“ ein phoniatrisch-logopädisches Gutachten vorliegen muss [131], stellen auch verschiedene Bildungseinrichtungen für PEU konkrete Anforderungen an das Gehör von Bewerbern. So ist beispielsweise eine Voraussetzung für den Eintritt in das Tonmeisterstudium der Zürcher Hochschule der Künste eine Normakusis im Reintonaudiogramm (Hörschwelle < 20 dB HL in den Frequenzen 125 Hz–8 kHz). Auch vor Antritt einer Ausbildung zur Logopädin oder zum Sonar-Techniker müssen audiologische Anforderungen erfüllt sein [70, 131]. Mit Vorteil findet zu Beginn der Ausbildung bzw. der Berufstätigkeit eine umfassende ohrenärztliche Konsultation statt, welche eine Erhebung der Ohranamnese, einen HNO-Status einschließlich einer Ohrmikroskopie sowie die Durchführung eines Reintonaudiogramms beinhaltet. Im Rahmen einer frühzeitigen ärztlichen Kontaktaufnahme des PEU im Sinne einer Vorsorge besteht auch die Möglichkeit, bereits eine ohrenärztliche Anlaufstelle auszuwählen, wo Erfahrung im Umgang mit PEU besteht und die im Bedarfs- bzw. Notfall aufgesucht oder mindestens kontaktiert werden kann.

Eine Normakusis in der Reintonaudiometrie ist in der Regel definiert als durchschnittlicher Hörverlust von weniger als 20 dB HL bis 25 dB HL in mehreren Hauptsprachfrequenzen (500 Hz–4 KHz) [103, 116, 117]. Eine solche Definition vermag jedoch insbesondere bei PEU zu kurz zu greifen. Der kleinste wahrnehmbare Schallpegelunterschied für reine Töne liegt unter 1 dB, wobei die höchste Sensitivität des Gehörs für Schallpegelunterschiede zwischen 2 kHz und 5 kHz liegt [121]. Weiter nimmt der kleinste wahrnehmbare Schallpegelunterschied mit zunehmendem Schallpegel ab, sodass bei gewissen Bedingungen ein Schallpegelunterschied von 0,2 dB wahrnehmbar ist [121]. Wie bereits erwähnt, ist darüber hinaus bekannt, dass PEU überdurchschnittliche auditive Wahrnehmungsfähigkeiten besitzen [58, 87]. Vor diesem Hintergrund ist es verständlich, dass für einen PEU bereits ein Hörverlust, welcher noch als Normakusis klassifiziert würde, als einschränkend empfunden wird, was insbesondere bei asymmetrischer Ausprägung der Fall ist [46]. So muss z. B. ein Tonmeister in der professionellen Abmischung von Musik auf minimale Unterschiede auch unterhalb von 1 dB achten, wobei von spezialisierten Tonmeistern das Erkennen von Unterschieden bis 0,1 dB erwartet wird [56]. Im Umgang mit PEU soll das Konzept der Normakusis im Reintonaudiogramm entsprechend informiert vermittelt werden. Dabei muss jedoch einschränkend auf die Messunsicherheit des Reintonaudiogramms von rund 10 dB hingewiesen werden [26]. Weiter soll beachtet werden, dass das Reintonaudiogramm verschiedene Formen von Einschränkungen des Gehörs nicht abbildet, so z. B. den „hidden hearing loss“ (s. Abschnitt „Lärm“) [67]. Weiter bildet das Reintonaudiogramm die Fähigkeit zur Verarbeitung komplexer, überschwelliger Schallinformation nur unzureichend ab, welche aber insbesondere bei PEU zentral ist [70]. Insofern soll die Indikation zu weiteren audiometrischen Untersuchungsverfahren großzügig erfolgen, z. B. zur Messung transitorisch evozierter oder distorsiv produzierter otoakustischer Emissionen sowie einer Sprachaudiometrie [31, 46]. Diese Untersuchungen können auf eine relevante Beeinträchtigung des Gehörs hinweisen, noch bevor diese im Reintonaudiogramm sichtbar wird [9, 22, 34]. Transitorisch evozierte otoakustische Emissionen wurden am Beispiel von normalhörenden Sängern bei PEU als Methode zur Risikobestimmung für einen zukünftigen, manifesten musikinduzierten Hörverlust vorgeschlagen [47], wobei diese Resultate noch nicht in weiteren Studien validiert wurden. Die Sprachaudiometrie kann in Ruhe oder – zur Früherkennung von Hörstörungen – im Störgeräusch durchgeführt werden [88]^2^. Eine Hochfrequenzaudiometrie (bis 20 kHz) kann zur weiteren Klärung einer subjektiven Schwerhörigkeit erfolgen, wobei jedoch die primäre Indikation zu dieser Untersuchung bei der Tinnitusdiagnostik insbesondere jüngerer Patienten besteht [111]. Die Hochfrequenzaudiometrie ist im Gegensatz zur konventionellen Reintonaudiometrie bis 8 kHz sensitiver, um einen Hörverlust zu finden, und hat somit möglicherweise auch bei der Früherkennung einer lärmbedingten Schwerhörigkeit eine Bedeutung, welche sich nicht nur bei 3 kHz bis 6 kHz, sondern auch bei 11 kHz bis 14 kHz manifestieren kann [10]. Weitere psychoakustische Tests mit möglicher spezieller Anwendung bei PEU stellen z. B. die „Gap Detection“, Mithörschwellen oder Tonhöhenunterscheidung dar.

Zusätzlich zur audiometrischen Untersuchung soll bei PEU wie erwähnt mindestens einmal eine vorsorgende ohrenärztliche Untersuchung erfolgen. So können auch beispielsweise die Anatomie des Gehörgangs sowie die Integrität des Trommelfells beurteilt werden, was für eine selbstständige, sichere und effiziente Reinigung des Gehörgangs eine Rolle spielt (s. Abschnitt „Cerumen obturans“).

## Spezielle Aspekte von Ohrerkrankungen und deren Therapie

Im Folgenden werden ausgewählte spezielle Aspekte von Ohrerkrankungen und deren Therapie bei PEU kursorisch besprochen. Bereits im Rahmen ihrer Ausbildung sollen PEU über Ohrerkrankungen informiert werden mit dem Ziel, Abklärung und Therapiekonzepte wichtiger Ohrerkrankungen nachvollziehbar zu machen.

### Cerumen obturans

Die Frage nach der korrekten Gehörgangsreinigung ist ein häufiges Thema in der Betreuung von PEU. Da eine falsch durchgeführte Gehörgangsreinigung erhebliche Gefahren für das Gehör birgt [100], kommt einer korrekten Gehörgangsreinigung bei PEU eine besondere Rolle zu. Darüber hinaus haben PEU, welche oft Gehörschutz oder Einsteck-Kopfhörer im Gehörgang tragen, ein erhöhtes Risiko für eine vermehrte Ansammlung vom Cerumen [102]. Grundsätzlich benötigt nicht jede Person reinigende Maßnahmen für den Gehörgang. Falls es tatsächlich zu einer erhöhten Zerumenproduktion und -ansammlung kommt, kann in erster Linie eine selbstständige Reinigung durch Spülung mit lauwarmem Wasser und Zerumenolytika wie beispielsweise einer 3%igen Wasserstoffperoxidlösung erfolgen, welche zusätzlich desinfizierende Eigenschaften aufweist [100]. Von jeglichen Instrumenten oder Objekten, z. B. Wattestäbchen, Haarnadeln oder „Ohrkerzen“, welche selbstständig in den Gehörgang eingeführt werden, ist aufgrund der oft kontraproduktiven Wirkung sowie einer erheblichen Verletzungsgefahr dezidiert abzuraten [32, 100, 101]. Um eine sichere selbstständige Anwendung sowie eine suffiziente Instruktion zu gewährleisten, empfiehlt sich vor einer regelmäßigen selbstständigen Reinigung des Gehörgangs und insbesondere bei Anwendung von Zerumenolytika eine HNO-ärztliche Untersuchung. Falls die selbstständig durchgeführten Maßnahmen nicht ausreichen, soll eine Ohrreinigung durch einen Ohrenarzt erfolgen, was bei Bedarf in regelmäßigen Intervallen durchgeführt werden kann [100]. Insbesondere bei PEU ist dabei zu bedenken, dass Ohrsauger äußerst zurückhaltend eingesetzt werden sollen, da dabei ein Spitzenschalldruck von bis zu 146 dB (A) auftreten kann [69].

### Chronische sensorineurale Schwerhörigkeit und Tinnitus

In den letzten Jahren wurde zunehmend klar, dass Lärm das Gehör bei deutlich schwächeren Schallpegeln dauerhaft schädigen kann als zuvor angenommen (s. Abschnitt „Lärm“). Während es bei Musikern im Umfeld von unverstärkter und verstärkter Musik zahlreiche Studien gibt, welche eine hohe Prävalenz von Hörstörungen einschließlich Tinnitus zeigen [31, 45], ist die Studienlage bei weiteren PEU dürftig. Lediglich für eine kleine Gruppe von Tonmeistern [79] sowie eine Kohorte von Sonar-Technikern [70] liegen Daten zum Gehör vor und für weitere Berufsgruppen wie Komponisten, Klavierbaumeister oder Dirigenten finden sich nur anekdotisch aktuelle Berichte [29]. Studien über Hörstörungen bei Komponisten entstammen hauptsächlich der musikgeschichtlichen Literatur [5, 35, 119]. In diesem Zusammenhang gilt es auch zu erwähnen, dass Musiker im Speziellen und wahrscheinlich PEU im Allgemeinen oft zurückhaltend an entsprechenden vorsorgenden Untersuchungen teilnehmen [63]. Dies ist möglicherweise auf eine Furcht vor einem (vermeintlichen) Stigma durch eine eventuelle Gehörschädigung zurückzuführen [63, 113].

Bereits ein subklinischer bzw. milder Hörverlust von 10 dB HL bis 20 dB HL kann sich stark auf die Schallwahrnehmung, insbesondere auf die Sprachverständlichkeit, sowie die neurokognitive Leistungsfähigkeit auswirken [42, 104]. Die auditorische Deprivation kann weiter zu Tinnitus führen, welcher nicht immer mit einer tonaudiometrisch fassbaren Schwerhörigkeit einhergehen muss [65]. Darüber hinaus kann auch eine nur diskrete Schwerhörigkeit dramatische Folgen für die Wahrnehmung hochkomplexer Schallinformation wie Musik haben [108]. Eine Erhöhung der Hörschwelle durch eine sensorineurale Schwerhörigkeit führt zu einer verminderten dynamischen Breite des verbleibenden Gehörs, was zu einem schnelleren Lautheitsanstieg führt und von einer Hyperakusis begleitet sein kann [7]. Weiter führt eine sensorineurale Schwerhörigkeit zu einer Reduktion der Fähigkeit zur Frequenzdiskrimination, wobei bei den in der Regel zuerst von einer sensorineuralen Schwerhörigkeit betroffenen höheren Frequenzen mit rund 0,7 % der kleinste wahrnehmbare Frequenzunterschied besteht [41, 121]. Dies kann u. a. die Wahrnehmung von Obertonspektren früh verändern. Zusätzlich kommt es bei einer sensorineuralen Schwerhörigkeit zu einer Verschiebung der maximalen Basilarmembranauslenkung in Richtung der Cochleabasis [18, 75]. Somit verschiebt sich die maximale Erregung von Hörnervenfasern zu tieferen Frequenzen, was auf zellulärer Ebene durch einen Verlust der äußeren Haarzellen und damit einer Verminderung der mechanischen Frequenzselektivität der Basilarmembran erklärt werden kann [18, 48]. In unserer Erfahrung kann bei PEU – insbesondere bei Vorliegen eines absoluten Gehörs – ein solches in Richtung tieferer Töne „verstimmtes“ Gehör eine starke Einschränkung in der Berufsfähigkeit bedeuten. Schließlich kommt es bei einer sensorineuralen Schwerhörigkeit zu Schalldistorsionen, deren Auftreten nur schwach mit dem Ausmaß der Hörminderung korreliert [89]. Zusammengefasst beeinträchtigt eine sensorineurale Schwerhörigkeit die Wahrnehmung akustischer Signale durch eine Reduktion der wahrgenommenen Lautstärke, einen schnelleren Lautheitsanstieg, eine reduzierte Frequenzdiskrimination, eine Frequenzverschiebung sowie Distorsionen. Dies hat umso einschränkendere Auswirkungen, je komplexer die eintreffende Schallinformation ist. Für PEU, welche in hoher Zuverlässigkeit Timbre, Lautstärke, Stimmung, Klanglokalisation oder Balanceeinstellungen beurteilen müssen, können solche Hörstörungen die Berufsausübung entscheidend einschränken.

Die Hörrehabilitation bei einer chronischen sensorineuralen Schwerhörigkeit erfolgt durch Hörgeräte. Eine Hörgeräteversorgung wird generell aufgrund der früh auftretenden Beeinträchtigung der Sprachverständlichkeit sowie der neurokognitiven Folgen einer Schwerhörigkeit bereits bei einem milden Hörverlust empfohlen [110]. Hörgeräte sind in erster Linie konstruiert, um durch verschiedene Methoden der Signalverarbeitung und -verstärkung die Sprachverständlichkeit zu verbessern. Komplexere Schallsignale wie Musik können durch Hörgeräte in der Regel nicht in einer Weise aufbereitet werden, dass ein natürlicher Höreindruck entsteht [15]. Diese Schwierigkeit beruht hauptsächlich auf den physikalischen Unterschieden zwischen Sprache und Musik [14, 17]: Unmittelbar dargebotene Musik hat ein deutlich weiteres und vielfältig gewichteteres Frequenzspektrum sowie einen größeren dynamischen Umfang als Sprache. Darüber hinaus hat Musik einen höheren Scheitelfaktor als Sprache, das heißt, die Spitzenwerte der Schallpegel weichen stärker vom gemittelten Schallpegel ab. Die Anpassstrategie, um mit einem Hörgerät die bestmögliche Wiedergabe von Musik zu erreichen, umfasst beispielsweise die Anhebung der für Sprache angepassten und oft auf etwa 85 dB SPL eingestellten Limitierung des Eingangssignals („peak input limiting level“) [14]. Zusätzlich soll die Frequenzbandbreite auf den Hörverlust angepasst werden [16, 92]. Schließlich kann eine Abschaltung der Systeme für die Rückkopplungsreduktion und die Geräuschreduzierung sowie die Verwendung eines omnidirektionalen Mikrofonmodus die Musikwahrnehmung verbessern [16]. Die Hörgeräteanpassung eines PEU soll bei einem spezialisierten Hörgeräteakustiker erfolgen, da die Wahrnehmung komplexer Schallinformationen wie Musik durch eine individuelle Anpassung entscheidend verbessert werden kann [15, 17, 93]. So scheint auch bei PEU schlussendlich eine zufriedenstellende Hörgeräteanpassung möglich [108]. An dieser Stelle sei auch darauf hingewiesen, dass in Deutschland die Rentenversicherung bei drohender Berufsunfähigkeit für die Mehrkosten eines Hörgeräts aufkommen kann, die über dem Festbetrag der gesetzlichen Krankenversicherung liegen.

### Obstruktive Tubenventilationsstörung

Eine über einen kürzeren Zeitraum bestehende obstruktive Tubenventilationsstörung ist nicht mit gefährlichen Folgeerscheinungen assoziiert, kann jedoch zu störenden Symptomen wie Schwerhörigkeit, Ohrdruck oder Tinnitus führen [99]. Als wichtige Differenzialdiagnosen dieser Symptome sind Drittfensterläsionen [27] sowie – auch bei isoliertem Ohrdruck – eine sensorineurale Schwerhörigkeit bzw. weitere Innenohrerkrankungen zu nennen [6, 85].

Eine obstruktive Tubenventilationsstörung kann in Kombination mit Veränderungen des atmosphärischen Umgebungsdrucks ein Barotrauma auslösen, was durch Verletzungen des Trommelfells oder der Rundfenstermembran zu persistierenden Hörstörungen führen kann [73]. Entsprechend sollen Flugreisen bei Unfähigkeit, einen Druckausgleich durchzuführen, unterlassen werden [73]. Da PEU oft wegen internationalen beruflichen Verpflichtungen in hoher Frequenz Flugreisen absolvieren müssen, ist ein sicheres und klares Vorgehen zum Management von Tubenventilationsstörungen zentral.

Als therapeutische Maßnahme soll in erster Linie ein „Training“ des Valsalva-Versuchs erfolgen sowie – insbesondere im Fall einer Flugreise – während Start- und Landephase regelmäßig gegähnt, geschluckt oder gekaut werden, um die Tubenöffnung zu begünstigen. Als medikamentöse Therapie sind systemisch abschwellende Substanzen hilfreich, z. B. oral verabreichtes Phenylephrin [21, 53]. Nasal applizierte abschwellende Medikamente sind zur Akuttherapie von Tubenventilationsstörungen wahrscheinlich wenig oder gar nicht wirksam [21, 53, 84], werden aber bei anfälligen Personen in der Praxis dennoch oft präventiv eingesetzt. Chirurgische Maßnahmen am Trommelfell (Parazentese, Einlage eines Paukenröhrchens) sind bei PEU äußerst zurückhaltend anzuwenden, da auch kleine Defekte des Trommelfells zu einem Hörverlust führen können [71, 94]. Zur Therapie einer *chronischen* obstruktiven Tubenventilationsstörung ist bei PEU eine Ballondilatation der Eustachischen Röhre der Einlage eines Paukenröhrchens vorzuziehen, auch wenn diese Therapie aktuell noch kontrovers diskutiert wird [37, 49].

### „Hearing when sick“

Für den „Professional *Voice* User“ stellt das Singen bei Krankheit („singing when sick“) eine Gefahr für das Stimmorgan dar und erfordert deshalb eine vorsichtige Risiko-Nutzen-Abwägung und ein spezialisiertes Management [76, 96]. „Krank“ bezieht sich dabei in der Regel auf leichte virale Infekte der oberen Atemwege („Erkältung“). Hierzu gibt es jedoch in Bezug auf das Gehör keine Daten, welche eine Gefährdung durch solche Infekte zeigen, auch nicht in Verbindung mit anderen schädigenden Einflüssen wie Lärm. Somit scheint ein leichter viraler Infekt der oberen Atemwege grundsätzlich keine Kontraindikation für das Ausüben beruflicher Tätigkeiten bei PEU zu sein. Virale Infekte der oberen Atemwege begünstigen allerdings eine Tubenventilationsstörung [11], welche entsprechend behandelt werden kann (s. Abschnitt „Obstruktive Tubenventilationsstörung“).

### Weitere Besonderheiten in der Therapie ausgewählter Ohrerkrankungen beim „Professional Ear User“

Die Abhängigkeit von einem überdurchschnittlich ausgebildeten Hörsinn bei PEU soll wie oben dargelegt in die Therapieentscheide bei diversen Ohrerkrankungen einfließen. Bei einer unkomplizierten akuten Otitis media wird zunehmend von einer primären Antibiotikagabe abgeraten, wobei Kriterien für eine sofortige Antibiotikagabe existieren, wie z. B. eine akute Otitis media auf einem letzthörenden Ohr [86, 105]. Falls eine akute Otitis media bei einem PEU auftritt, ist gegebenenfalls eine frühzeitige Therapie mit primärer Antibiotikagabe gerechtfertigt. Ein Hörsturz oder ein akut aufgetretener Tinnitus bei einem PEU rechtfertigt auch bei aktuell mäßiger Evidenz eine frühzeitige Therapie mit hochdosierten Kortikosteroiden, während grundsätzlich beim Hörsturz im Einvernehmen mit dem Patienten auch für einige Tage eine Spontanremission abgewartet werden kann [129].

In der Diagnostik und Therapie von PEU wird der Ohrenarzt weiter diverse Krankheitsbilder antreffen, welche schwierig einzuordnen sind. Grundsätzlich muss immer auch an die Möglichkeit einer organischen, jedoch objektiv nicht fassbaren Ohrerkrankung gedacht werden. Dabei ist zu bedenken, dass ein PEU durch eine Ohrerkrankung, insbesondere eine Schwerhörigkeit, deutlich früher und stärker gestört sein kann als ein Patient des Durchschnittskollektivs. Eine wichtige Differenzialdiagnose schwierig oder nicht fassbarer Beschwerden sind jedoch auch funktionelle, nichtorganische Ohrerkrankungen. Gelegentlich haben solche Erkrankungen ihren Ursprung in einer objektivierbaren Veränderung, die jedoch erst durch deren übermäßige subjektive Bedeutungsverstärkung ihre pathologische Auswirkung erfährt [8]. Gerade die Abhängigkeit von einem „perfekten“ Gehör vermag einen solchen Teufelskreis zu begünstigen. Funktionelle, nichtorganische Ohrerkrankungen können sich äußern durch jegliche Qualitätsänderungen des Gehörs, einen Hörverlust, ein Ohrdruckgefühl oder Tinnitus [8]. Eine verständnisvolle, informative Aufklärung und Beruhigung sind die zentralen Bestandteile in der Therapie von funktionellen Ohrerkrankungen, was insbesondere bei PEU aufgrund des technischen und künstlerischen Vorwissens sowie der Bedeutung des Gehörs für die Berufsausübung zeitaufwendig sein kann [8]. In schwereren Fällen soll eine psychologische oder psychiatrische Begleittherapie erwogen werden [8]. Funktionelle, nichtorganische Ohrerkrankungen sind mit psychischen Erkrankungen assoziiert [4, 8], welche gerade bei PEU mit hohem Leistungsanspruch und -druck eine erhöhte Prävalenz aufweisen [36, 112] und deshalb aktiv gesucht werden sollen.

## Programme zur Erhaltung der Ohrgesundheit beim „Professional Ear User“

Eine spezialisierte und umfassende Aufklärung zur Ohrgesundheit bei PEU sowie die Vermittlung von Anlaufstellen zur Prävention, Diagnostik und Therapie von Ohrerkrankungen sind zentral zur Umsetzung eines Programms zur Förderung und Erhaltung einer umfassenden Ohrgesundheit bei PEU. Eine Vielzahl von existierenden Einrichtungen und Programmen verfolgen ähnliche Ziele. So findet sich im deutschsprachen Raum z. B. das vielfach ausgezeichnete Freiburger Institut für Musikermedizin, welches die Prävention und Therapie von Hörstörungen bei Musikern als einen Schwerpunkt der Einrichtung nennt [123]. Vergleichbare Anlaufstellen finden sich in mehreren deutschsprachigen Ländern [124–126]. Im angloamerikanischen Raum besitzen verschiedene universitäre HNO-Kliniken ein „Musicians’ Hearing Center“ [127]. Bemerkenswert ist auch das „Musicians’ Hearing Health Scheme“ der britischen „Musicians’ Union“ [77]. Dieses subventionierte Programm ermöglicht Mitgliedern einen vereinfachten und vergünstigten Zugang zu spezialisierten Akustikern und Ärzten, ein „Ohr-Check-up“ einschließlich einer audiometrischen Untersuchung und einer Gehörgangsreinigung, die Anschaffung otoplastischer Gehörschutzmittel und schließlich eine regelmäßige Erinnerung an eine Verlaufsaudiometrie im Abstand von zwei Jahren.

Die bestehenden Programme und Maßnahmen sind fast ausschließlich für Musiker ausgelegt und richten sich kaum an andere Berufsgruppen, welche jedoch ebenfalls in hohem Maß auf ein gesundes Hörorgan angewiesen sind. Weiter gibt es kaum Programme, welche eine umfassende „Ohrgesundheit“ abdecken, da sie oft nur auf Lärm als Risikofaktor und Hörstörungen wie eine Schwerhörigkeit oder Tinnitus ausgerichtet sind. Im Aufbau eines entsprechenden Programms sehen wir jedoch kaum Gründe, dieses nicht aktiv auf weitere in einem professionellen Rahmen auf ein überdurchschnittliches Hörvermögen angewiesene (und entsprechend gefährdete) Personen auszudehnen. Dies bietet sich darüber hinaus im Rahmen von entsprechenden Vorlesungen oder Seminaren an Hochschulen geradezu an, wo gleichzeitig PEU verschiedener Berufsgruppen ausgebildet werden. Ein solches Programm soll neben Lärm auch über weitere Risikofaktoren für das Gehör wie Ototoxine oder Nikotin aufklären, Grundsätze der Gehörgangshygiene näherbringen, den Umgang mit häufigen Ohrproblemen wie Tubenventilationsstörungen zur Vermeidung von Komplikationen lehren und über ohrenärztliche Anlaufstellen informieren. Dies kann beispielsweise in entsprechenden Seminaren im Rahmen der Ausbildungslehrgänge der PEU vermittelt werden. Weiter kann in Kooperation mit den Hochschulen und Ausbildungsinstitutionen für PEU eine ohrenärztliche Anlaufstelle angeboten werden, welche bereits auch die vorsorgenden Untersuchungen durchführen könnte. Somit soll mit einem entsprechenden edukativen Programm und einer spezialisierten, ohrenärztlichen Sprechstunde eine ganzheitliche Ohrgesundheit in der Gesamtheit der Berufsgruppen gefördert werden, welche in professionellem Rahmen stark von einem optimal funktionieren Hörorgan abhängig sind.

## Empfehlungen und Fazit für die Praxis

- „Professional Ear Users“ (PEU) besitzen eine überdurchschnittliche auditive Wahrnehmungsfähigkeit und sind stark von dieser abhängig. Weiter besteht oft ein großes Wissen bezüglich der technischen und künstlerischen Seite von Schall. Somit bringen PEU spezielle Voraussetzungen und Anforderungen an die Prävention und Diagnostik von Ohrerkrankungen mit.
- PEU sollen möglichst früh im Laufe der Berufsfindung mit der Anatomie und Physiologie des Ohrs sowie mit präventiven Maßnahmen von Ohrerkrankung vertraut gemacht werden.
- Ein zu hoher Schalldruckpegel ist mit Abstand der wichtigste vermeidbare Risikofaktor für eine Schwerhörigkeit. Ein konsequenter Gehörschutz bei schädlichen Schallpegeln ist zentral für PEU.
- Eine ohrenärztliche Vorsorgeuntersuchung zu Beginn der Ausbildung sowie an das individuelle Risikoprofil angepasste regelmäßige audiometrische Untersuchungen sind wünschenswert.
- Eine normale Hörschwelle im konventionellen Reintonaudiogramm schließt eine Hörstörung nicht aus. Auch deshalb muss die Definition der „Normakusis“ bei PEU mit besonderer Zurückhaltung angewendet werden. Weiterführende Untersuchungen sollen großzügig eingesetzt werden.
- Bei PEU soll in der Diagnostik von Ohrerkrankungen eine erhöhte Sensitivität gegenüber Krankheitsbildern bestehen, welche in einem Durchschnittskollektiv als „subklinisch“ gelten können. Eine wichtige Differenzialdiagnose schwierig fassbarer Beschwerden sind jedoch auch funktionelle Ohrerkrankungen.
- Das Vorliegen eines PEU als Patient soll als Faktor in Aufklärungsarbeit, Betreuungsaufwand und Therapieentscheide bei Ohrerkrankungen einfließen.
- Eine spezialisierte und umfassende Aufklärung zur Ohrgesundheit sowie die Vermittlung von spezialisierten Anlaufstellen zur Prävention, Diagnostik und Therapie von Ohrerkrankungen sind zentral für PEU.

## Footnotes

1 Sonar-Techniker bedienen Sonar-Ortungssysteme, welche typischerweise in U‑Booten zur militärischen Unterwasserüberwachung eingesetzt werden [132]. Das Sonar-Ortungssystem produziert ein akustisches Signal, anhand dessen der Sonar-Techniker die Größe, Beschaffenheit, Distanz und Geschwindigkeit eines georteten Objekts beurteilen kann [70, 132].

2 Hierzu müssen Sprachtests mit hoher Messgenauigkeit verwendet werden, was z. B. für den Freiburger Einsilbertest nicht gegeben ist. Außerdem ist bei Anwendung eines Störgeräuschs mit einem Signal-Rausch-Abstand von +5 dB im Fall einer Normakusis im Tonaudiogramm kein aussagekräftiges Resultat zu erwarten. Notwendig ist ein Test im Störgeräusch mit steiler Diskriminationsfunktion, bei dem die Schwelle für ein Sprachverstehen von 50 % mit einem adaptiven Verfahren genau ermittelt werden kann.
